# Umbrella and basket trials in oncology: ethical challenges

**DOI:** 10.1186/s12910-019-0395-5

**Published:** 2019-08-23

**Authors:** Karolina Strzebonska, Marcin Waligora

**Affiliations:** REMEDY, Research Ethics in Medicine Study Group, Department of Philosophy and Bioethics, Jagiellonian University Medical College, ul. Michałowskiego 12, 31-126 Krakow, Poland

**Keywords:** Umbrella trial, Basket trial, Master protocol, NCI-MATCH, Lung-MAP, Scientific validity, Risk-benefit balance, Informed consent, Ethics

## Abstract

**Background:**

Novel precision oncology trial designs, such as basket and umbrella trials, are designed to test new anticancer agents in more effective and affordable ways. However, they present some ethical concerns referred to scientific validity, risk-benefit balance and informed consent. Our aim is to discuss these issues in basket and umbrella trials, giving examples of two ongoing cancer trials: NCI-MATCH (National Cancer Institute – Molecular Analysis for Therapy Choice) and Lung-MAP (Lung Cancer Master Protocol) study.

**Main body:**

We discuss three ethical requirements for clinical trials which may be challenged in basket and umbrella trial designs. Firstly, we consider scientific validity. Thanks to the new trial designs, patients with rare malignancies have the opportunity to be enrolled and benefit from the trial, but due to insufficient accrual, the trial may generate clinically insignificant findings. Inadequate sample size in study arms and the use of surrogate endpoints may result in a drug approval without confirmed efficacy. Moreover, complexity, limited quality and availability of tumor samples may not only introduce bias and result in unreliable and unrepresentative findings, but also can potentially harm patients and assign them to an inappropriate therapy arm. Secondly, we refer to benefits and risks. Novel clinical trials can gain important knowledge on the variety of tumors, which can be used in future trials to develop effective therapies. However, they offer limited direct benefits to patients. All potential participants must wait about 2 weeks for the results of the genetic screening, which may be stressful and produce anxiety. The enrollment of patients whose tumors harbor multiple mutations in treatments matching a single mutation may be controversial. As to informed consent – the third requirement we discuss, the excessive use of phrases like “personalized medicine”, “tailored therapy” or “precision oncology” might be misleading and cause personal convictions that the study protocol is designed to fulfill the individual health-related needs of participants.

**Conclusions:**

We suggest that further approaches should be implemented to enhance scientific validity, reduce misunderstandings and risks, thus maximizing the benefits to society and to trial participants.

## Background

The current move to genomics changes the diagnostic information needed for treatment and belongs to a novel concept of personalized or precision medicine [1]. Its aim is to use tailored therapies to target specific genetic changes that cause the tumor to develop. Thanks to the understanding of each person’s cancer at the molecular level, it can be possible to adjust the appropriate drug and dose, thus maximizing the benefit of targeted treatment for the individual [1, 2].

Development of new medicines that work only on a specific type of malignancy, or more specifically, on a particular genetic abnormality, requires new approaches to conducting clinical trials. The American Food and Drug Administration (FDA) encourages implementing innovative clinical trial designs like umbrella trials or basket trials as they give hope for better treatments and very effective drugs, but according to us, they also present ethical challenges. For instance, if we consider a classic approach by Emanuel et al. [3] at least three ethical requirements must be analyzed: scientific validity, favorable risk-benefit ratio and informed consent. We narrow down our discussion to basket and umbrella trials in oncology, which is a limitation of our study, but some of our considerations can possibly be applied to other contexts.

Before we refer to the main considerations, we briefly describe these types of trials and give an example of each in order to give a basis for further reflections.

### Master protocols: basket trials and umbrella trials

**Fig. 1:**
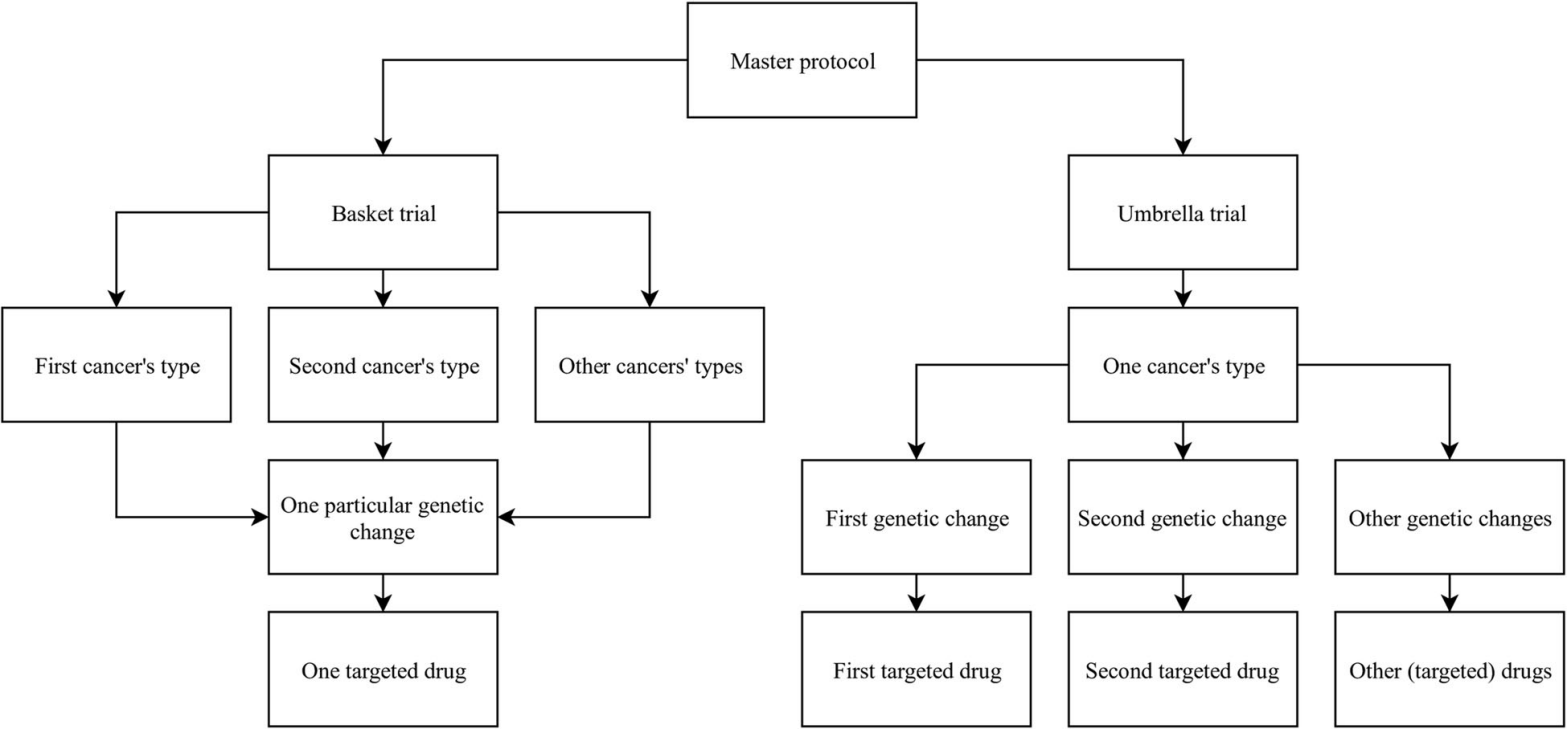
General schema dividing master protocol into a basket or an umbrella trial. A basket trial enrolls patients with different cancer types but sharing one common molecular alteration. They receive one treatment. An umbrella trial enrolls patients sharing the same cancer type but different molecular alteration. The treatment is adjusted to the specific target

Master protocol is a research process designed to test multiple targeted therapies in small sub-trials or cohorts [2, 4]. Patients with cancer are assigned to an arm of a clinical trial based on their targeted abnormality found in the tumor [2]. A flexible structure allows for adding more arms over time and the ineffective ones can be closed, without writing a new protocol [5]. We can distinguish two main types of master protocols: a basket trial and an umbrella trial (Fig. 1).

### Basket trial

A basket trial enrolls patients with any cancer type (e.g., colon, breast, lung and others) but who share the same genetic abnormality (Fig. 1) [6]. Generally, it can be a single- or multiple-arm trial, in which one arm is a separate “basket” that assigns small cohorts of patients and focuses on testing one treatment against a specific target, regardless of disease types [4]. It allows testing of a new drug against various cancers at the same time. The term “basket” refers to the fusion of potentially different cancers (according to the common classification by the body organ where they begin [6] or by their histological type of origin [7, 8]) into one similar disease at the molecular level.

**Fig. 2:**
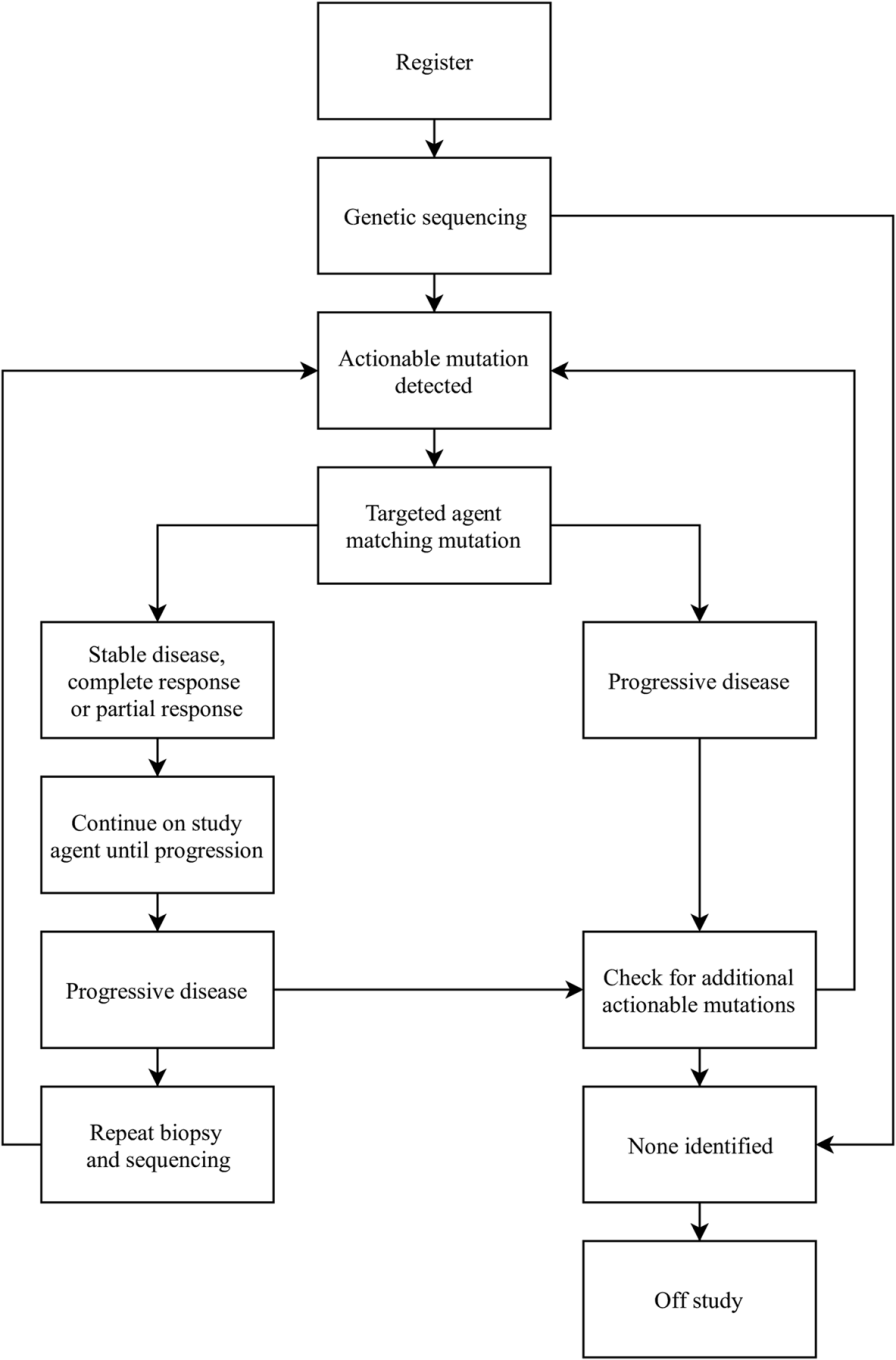
The schema of NCI-MATCH study design. The biopsy material derived from registered patients is characterized for specific pre-defined mutations via genetic sequencing. If an actionable mutation is detected, patients are assigned to 1 of 30 treatment sub-protocols. Those experiencing disease progression or serious adverse events undergo review of their previous biopsy results or undergo another biopsy to search for alternative treatment [10]

A common example of a basket trial is an ongoing phase II NCI-MATCH (Molecular Analysis for Therapy Choice, NCT02465060) trial launched in 2015 by the US National Cancer Institute (NCI) [9]. The general schema of this study is shown in Fig. 2.

Patients with any advanced solid tumors, lymphomas or myeloma who have progressed on standard of care or for whom there is no standard treatment may be eligible to register to NCI-MATCH trial [9]. After enrollment, samples obtained from a biopsy are screened to determine whether the tumors contain specific genetic changes that can be matched to the drugs being studied in the trial. Then, patients are assigned to the treatment arms [11]. If more than one genetic abnormality is found, the patient is assigned to the arm that is the most promising for a direct therapeutic benefit or to the arm that starts the earliest to provide treatment as soon as possible [4]. If the disease progresses, the patient may be treated with other drugs tested in the study or undergo a repeat biopsy [5]. The trial’s primary endpoint is the objective response (OR) rate, which includes complete or partial response to treatment, and the secondary endpoint is progression-free survival (PFS) at 6 months of treatment with targeted study agent [9].

The initial results for 3 NCI-MATCH arms are summarized in Table 1. The enrollment in this trial is dynamically expanding and new arms are open for accrual.

**Table 1:** Summary results of three NCI-MATCH sub-studies [12–14]

Sub-study	Pts enrolled N	Pts evaluable for response N	PR (%)	SD (%)	PD (%)	PFS6 rate (%)	Pts evaluable for toxicity N	AEs (%)	Grade 3/4 AEs (%)	Comments
Arm W: Pts with FGFR1–3 mutation or translocation receive FGFR Inhibitor AZD4547 [12]	52	41	5	51	44	17	49	80	49	Failed to meet its primary endpoint
Arm Q: Pts with HER2 amplification receive trastuzumab emtansine [13]	N/R	37	8.1	43	N/R	24.8	N/R	N/R	N/R	Failed to meet its primary endpoint
Arm I: Pts with PIK3CA mutation without RAS mutation or PTEN loss receive taselisib [14]	65	N/R	0	N/R	N/R	27	N/R	N/R	N/R	Failed to meet its primary endpoint; Co-occurring mutations were detected in 67% of tumors; 11% of pts. discontinued taselisib because of toxicity

*AEs* Adverse events, *N/R* Not reported, *OR* Objective response, *PD* Progressive disease, *PFS6* Progression-free survival at 6 months of treatment, *PR* Partial response, *Pts* Patients, *SD* Stable disease

### Umbrella trial

An umbrella trial enrolls patients with one cancer type but with different genetic changes within each tumor (Fig. 1). It consists of many small sub-trials to test multiple drugs simultaneously in one large trial [2, 6]. Patients receive different targeting treatments matched to their genetic aberration. The term “umbrella” refers to separation of one alleged cancer into many sub-cancers depending on their molecular features. There is also a “default arm” which assigns patients without a specific marker to receive standard treatment [6].

**Fig. 3:**
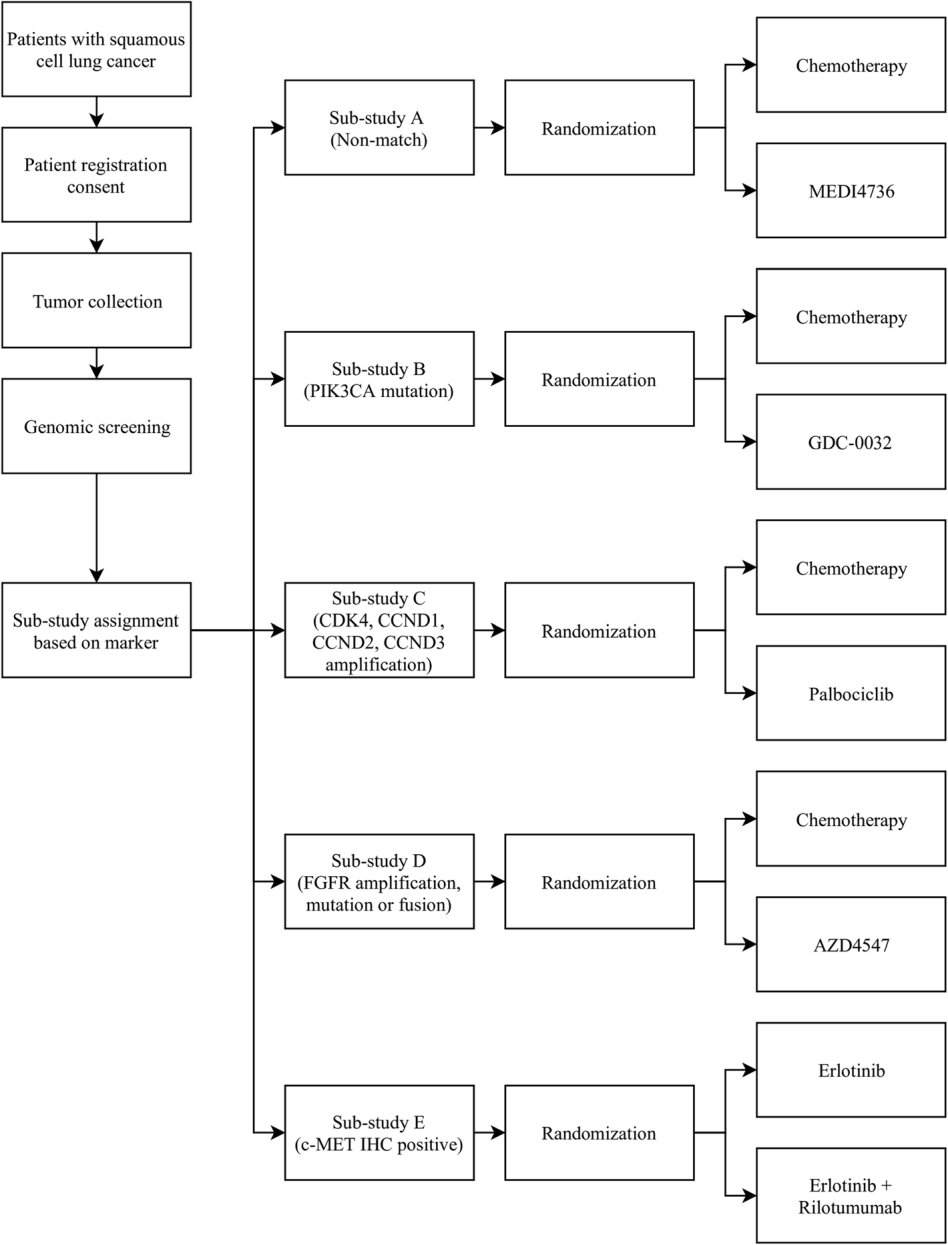
The schema of the Lung-MAP study design with five initial sub-studies. Adult patients with recurrent or metastatic squamous cell carcinoma (SCC) after progression on first-line platinum-based chemotherapy could be eligible to register in the Lung-MAP trial. After signing an informed consent, their archival or fresh tumor biopsy sample was screened for genetic aberrations. Results of genomic testing were returned within 16 days. Then, all patients were originally assigned to one of five sub-studies and they were randomized to receive either standard of care or a specific agent tailored to their alteration. One of these sub-trials was called a “non-match” sub-study and it enrolled patients whose tumors did not harbor any of the genetic aberrations tested in other sub-studies [15, 16]

The Lung-MAP: S1400 Phase II/III Biomarker-Driven Master Protocol for Second Line Therapy of Squamous Cell Lung Cancer (NCT02154490) is an example of an ongoing umbrella study sponsored by Southwest Oncology Group (SWOG) [15]. Its general schema at the time of opening in 2014 with five original treatment sub-trials is presented in Fig. 3.

Adult patients with advanced squamous cell cancer (SCCA) of the lung who progressed on first-line platinum-based therapy could be eligible to enroll into the Lung-MAP trial. After genomic screening of their tumor sample, they were initially assigned to one of the five independent sub-studies (four targeted therapies and one non-match therapy) and randomized to receive either an investigational drug or standard treatment. If more than one genetic change was found, they were assigned to a sub-trial based on a pre-defined algorithm that balanced accrual among sub-studies. If no actionable marker was detected, they were enrolled to the non-match sub-trial, allowing all eligible patients to be treated [16]. The trial’s primary endpoint was response rate [17]. The decision to either close a sub-study or move to a phase III registration trial was based on the interim analysis of each phase II sub-trial [16].

Since June 16, 2017 all of these five original sub-studies have been closed to accrual and their interim results are presented in Table 2 [17]. Randomization has been amended [17] and currently, four new sub-studies (S1400F, S1400G, S1400I and S1400K) are a part of the Lung-MAP trial and are open for accrual.

**Table 2:** Summary results of five initial Lung-MAP sub-studies [17]

Sub-study	ClinicalTrials.gov identifier	Final accrual	Response rate to investigational therapy N (%)	Comment
S1400A (non-match)	NCT02766335	Total: 116	11 (16)	Administratively closed to enable activation of a new non-match study
		Chemotherapy: 38		
		MEDI4736: 78		
S1400B	NCT02785913	Total: 39	1 (4)	Failed to meet its primary endpoint,closed at interim futility analysis
		Chemotherapy: 8		
		GDC-0032: 31		
S1400C	NCT02785939	Total: 54	2 (6)	Failed to meet its primary endpoint,closed at interim futility analysis
		Chemotherapy: 17		
		Palbociclib: 37		
S1400D	NCT02965378	Total: 45	2 (7)	Failed to meet its primary endpoint, closed at interim futility analysis
		Chemotherapy: 10		
		AZD4547: 35		
S1400E	NCT02926638	Total: 9	N/A	Closed early due to discontinuation of development of rilotumumab
		Erlotinib: 5		
		Erlotinib + Rilotumumab: 4		

## Main text

### Scientific validity

Scientific validity of the research is one of the essential ethical requirements [18] as the overarching goal of a clinical trial is to provide evidence that can support clinical decision-making [19]. Here, we outline some of the major challenges referring to scientific validity in basket and umbrella trials.

The first issue that can be a threat to scientific validity is the design of a treatment that matches only a single mutation, while tumors may harbor multiple mutations at a time. Cancer’s heterogeneity can be distinguished not only within the primary tumor (intratumoral heterogeneity), but also between the primary tumor and its metastases (intertumoral heterogeneity) and between patients (interpatient heterogeneity) [4, 20, 21], which indicates that every tumor is unique. Thus, focusing only on molecular therapy targeting single mutation without considering the complexity of tumor biology, may introduce bias. It is unclear how many patients in NCI-MATCH and Lung-MAP trials are diagnosed to harbor more than one mutation. There may be more patients that only partially match the intervention (harbor multiple genetic changes) than the ones that totally match (harbor only one genetic change). Moreover, in a trial with randomization, like in the Lung-MAP trial, patients in both arms (experimental and standard of care) should harbor similar genetic changes within a tumor to be comparable and represent the same patient population. If the complexity and heterogeneity of tumors are neglected, the result may be a treatment failure and the impossibility to produce scientifically reliable findings.

The flexible structure of basket and umbrella trials allows for testing multiple interventions simultaneously, closing ineffective ones and opening new ones without writing a new protocol, which saves time and financial resources. Nevertheless, after closing a treatment, the results of the trial sub-study should be published as soon as possible as a full journal publication because the profound results of completed clinical trials are crucial for decision making in evidence-based medicine and inform future research. Unfortunately, summary results of NCI-MATCH and Lung-MAP sub-studies are incomplete. For example, it is hard to say in which arm of the five sub-studies in the Lung-MAP study subjects benefitted more (intervention or standard of care), because response rates in the standard of care arms have not yet been published (Table 2). Since we do not know whether responses are higher or lower in the standard of care arm, we claim that the risk of publication bias is the second challenge of the scientific validity requirement.

The flexibility enables also for changes in the protocol. Giving the exact example is the initial design of the Lung-MAP trial as a study including randomization to a control arm, which was further modified during the trial into single-arm study [17]. There is no explanation why it was changed. This can mean that the design was flawed from the beginning and no one had foreseen that. The freedom for unexplained modifications in the protocol is a third serious threat to scientific validity.

The main advantage of basket trials is that patients with rare cancers have the opportunity to be enrolled into the study. For example, in the NCI-MATCH trial, about 61% of patients have less common tumors [22]. However, a fourth serious issue referred to scientific validity is insufficient patient accrual to treatment arms, which may affect statistical methods and power and oppugn the reliability of the findings. For instance, in the NCI-MATCH trial only 8 of the 30 sub-studies reached the minimum patient accrual goal of 35 [23]. A research that cannot enroll sufficient subjects cannot generate valid scientific knowledge and is unethical [3]. Furthermore, waiting for more patients that harbor a specific mutation prolongs the study and delays the publication of trial results. There are also doubts whether drugs tested on an insufficient number of patients could be approved without confirmed efficacy based on surrogate endpoints which are considered low-grade evidence [24].

### Benefits and risks

Another important ethical requirement of conducting clinical trials is a favorable risk/benefit ratio, which is met when: 1) the risk for participants is minimized; 2) the expected benefits are maximized; and 3) the possible benefits to participants and society outweigh or are proportional to the risks associated with participation in the study. The risk-benefit proportionality criterion considers the fundamental ethical principles of non-maleficence and beneficence. It also serves as a protection for participants against their exploitation [3].

There are three types of possible benefit in clinical trials: aspirational, direct and collateral [25]. Aspirational benefit is the benefit to society and to future patients, which arises from the results of the study [25]. Novel clinical trials serve as exploratory trials of both tumor and pathways and they can gain important knowledge, which can be used in future trials to develop effective therapies. Thus, it is extremely important that the results of a trial are published after each sub-trial completes. Moreover, the aspects mentioned before in the scientific validity section: the heterogeneity of tumors harboring multiple mutations, problems with sufficient patient accrual, risk of publication bias may generate unreliable and unpowered findings, which negatively influences the aspirational benefit, wastes resources and negatively affects decision making in medicine [18].

Direct benefit is the benefit to research subjects arising from receiving the intervention being studied [25]. In cancer trials an optimal direct benefit could be the one achievable in patient-centered outcomes, such as overall survival (OS) and/or quality of life (QoL) [26]. However, in basket and umbrella trials the main measured outcomes are surrogate endpoints such as: progression-free survival (PFS), time to progression (TTP), tumor shrinkage, the percentage of patients responding to a drug or biomarkers that can predict clinical outcomes like survival. Surrogate endpoints substitute clinically meaningful endpoints and they are used to indicate whether treatment works. The use of surrogate endpoints in certain phases of research is justified [2, 24, 26] and their advantage is that they can yield information about the effect of a drug more rapidly than long-term clinical outcomes. Unfortunately, there is recently mounting evidence illustrating that surrogate endpoints do not necessarily translate to patient-centered outcomes [24, 27–29]. This also means that the direct benefit achieved by participants of umbrella and basket trials is arguable.

Other data suggest that the minority of patients who have been treated with genome-driven therapy benefited to date [30]. However, other findings show that overall response rate in all published basket trials in cancer medicine until March 2018 was 25%, which seems very promising [31]. But published results of NCI-MATCH and Lung-MAP trials are not equally promising. In NCI-MATCH, all three arms (Table 1) failed to meet their primary endpoint (25% or more of the patients whose tumors have a complete or partial response to treatment). Nevertheless, there were 17% or more patients with prolonged stable disease. Similarly, in the Lung-MAP study (Table 2),v all of the five subprotocols failed to meet their primary endpoint (25% or more of the patients whose tumors have a complete or partial response to treatment). Surprisingly, participants in the non-match sub-study benefitted more than the ones who matched, which may indicate that the treatment adjusted to a genetic change is less effective than the treatment that does not match.

The third type of benefit – “collateral” benefit, refers to benefit arising from being a research subject, like free medical care or the personal gratification of altruism [25]. This kind of benefit is easily discernible in basket and umbrella trials as thousands of patients may be screened and find out more about their disease and take part in the research process. However, not every patient can be screened as some tumors are too small to be collected for research purposes [32]. Even if they are eligible to be screened, the genetic profiling may fail to detect any actionable mutation. If there is an alteration, sometimes there is a lack of standard treatment or ongoing trial to offer. For example, in the Lung-MAP trial every patient can be treated, because a “non-match” study exists, unlike in the NCI-MATCH study, in which patients without actionable mutations are out of the study. In the NCI-MATCH trial 5963 tumors were screened for 30 treatment arms from opening in 2015 thru 16th July 2017 and in 5546 (93%) the assay was successful. Among them, 998 patients were assigned to matched interventions, but only 689 (69%) enrolled in all treatment arms, which gives 12% of all screened patients who finally started the therapy [22].

Despite the fact that basket and umbrella trials allow for recruitment of patients with rare malignancies, which can be considered as their main advantage, it is unclear why only some provide a “default arm” and some do not. It may be worth to explore various rare tumors and enable patients with no standard treatment options to be part of the research and contribute to gaining knowledge that can be used to develop new therapies.

There are many different types of risk or harm to research subjects, such as: physical, psychological, economic, legal or dignitary [33]. The first two types - physical and psychological, may appear at different stages during the research process of basket and umbrella trials. In these studies, some invasive procedures e.g., biopsy or surgical resection are required to collect an adequate tumor sample for evaluation via genetic profiling. Patients only take part in this research process if it is feasible and supposedly safe to obtain tumor material for molecular and genomic studies [32]. A tumor biopsy may be considered safe, but it can be stressful and uncomfortable for volunteers [34]. There is always a possibility of complications, especially when dealing with patients who progressed after chemotherapy and their organisms are weak. In turn, Overman et al. [35] raise issues with underreporting of results derived from research biopsies and provide recommendations to improve such reporting. Biopsy findings are particularly important in basket and umbrella trial designs as they are crucial to include or exclude a large number of potential participants from further steps of the study and they are supposed to generate knowledge and justify the risk.

While large volumes of tumor tissue are required for assessment and optimal diagnosis, sometimes depending on the localization of the lesion, it is challenging or even impossible to obtain a sufficient amount of tissue. Moreover, a biopsy material may be of low quality and/or not accurately capture the complete genomic landscape of the patient’s cancer, so only a limited geographical region of tumor is analyzed [21]. These both may result in mismatching and assigning the patient to inappropriate study arm and therefore, pose harm. Allocation to a substandard treatment either after a biomarker test result or after randomization is the most serious problem in basket and umbrella trials. They are designed to test one targeted treatment against one specific abnormality found in the patient’s tumor but there are instances where more than one genetic change is diagnosed. Such precarious single-target treatment may be insufficient and not match the entire heterogeneous tumor, resulting in disease progression. In this case, the intervention that targets the whole tumor may be more beneficial to the participant.

Consider the case of two patients enrolled to e.g., S1400B sub-study (Table 2): one with a single genetic defect, and the other with multiple genetic changes clearly identified, who are randomized to receive either chemotherapy or experimental intervention. If the first one is allocated to the chemotherapy arm and the second to the experimental arm it may not only be disadvantageous for them to obtain direct therapeutic benefit but also for acquiring reliable and reproducible results.

The process of screening and genome sequencing must be efficient and quick. From the patient’s perspective, the screening delay means being untreated for 2 weeks while awaiting results. For some oncology patients, 14 or 16 days is not many, for others every single day may be crucial and full of hope, stress and uncertainty, whereas some may even not survive to be enrolled.

Other risks in basket and umbrella trials are the same as in other areas of medicine. For example, there is a problem how to cope with any incidental findings found during genetic sequencing, which contain important health or reproductive information about participants [32]. Moreover, the recruitment of thousands of participants generates a huge amount of data that must not only be rapidly processed, but also reliably and safely stored, so that undesirable people have no access to it.

The ethics of research with human beings demands that patient-subject burdens are redeemed by gains in generalizable knowledge. Taking into consideration both risks and benefits, we claim that there is a low chance of direct therapeutic benefit to participants due to the major flaw in the design of these trials, which include patients with multiple genetic aberrations and test treatments against a single aberration, which can harm patients and may fail in gaining reliable findings. If this flaw is reduced by including patients with only one actionable mutation, then the risks can be justifiable.

### Informed consent

Informed consent is the cornerstone ethical principle of biomedical research [19] and it can be challenged in oncology clinical trials, including basket and umbrella trials. The perception of patients with life-threatening diseases is often affected by a desperate hope for the therapeutic benefit. They may understand the nature of the research process, but sometimes a clinical trial is their last hope and the last chance for any therapeutic benefit. This is where therapeutic misunderstanding can appear [36].

One such misunderstanding, the main ethical concern in cancer research, is a therapeutic misconception defined as “the belief that the purpose of a clinical trial is to benefit the individual patient rather than to gather data for the purpose of contributing to scientific knowledge” [37]. This tendency is common in all types of trials and can affect both patients willing to participate in a trial, and investigators/physicians who feel a therapeutic duty to deliver the best medical care to patients.

In basket and umbrella trial designs patients are divided into two groups – those who “match” and “not match” the experimental treatment. In the NCI-MATCH study, only the group that “matches” remains in the trial and receives intervention and the rest are excluded from the trial, while in the Lung-MAP study both those who match and do not match are treated. We think that the group that “matches” may have overestimated expectations and personal conviction that intervention is directly adjusted to each individual, although all subjects are treated according to the protocol. The same goes for another form of misunderstanding called “therapeutic misestimation”, which is described as “misunderstanding the probability of direct benefit or harms that may result from participating in research” [38] - subjects that “match” may overestimate potential chances to receive benefit and underestimate the risks, because they are “the chosen ones”. The problem of such misunderstandings can be intensified in trials with “non-match” sub-study without blinding of participants where everyone knows to which sub-trial they were assigned. In these trials, subjects in arms that “matched” may be 100% confident that they receive the treatment that best matches their disease and they will surely benefit, while patients in “non-match” may believe that the therapy they receive does not work at all, which can pose problems with reporting adverse events and assessing therapy efficacy.

What can even deepen therapeutic misunderstandings among those who want to participate in novel precision medicine trials is the excessive use of phrases like “personalized” or “individualized medicine”, and “tailored” therapy. Such terminology can be misleading, and falsely indicate that the trial’s goal is to provide personalized care with regard to the patient’s best interest standard and direct therapeutic benefit. We propose to avoid such phrases in master protocols and use more generalizable ones, such as “therapy based on genomics”.

We suggest that the problem of misunderstandings in precision medicine trials should be evaluated and reduced in view of giving valid informed consent. Without fully understanding the purpose of the trial as well as the real consequences of participation, a subject’s informed decision to take part in research is ethically challenged. Additionally, patients should be provided with information about the prospect of benefitting from the screening process while considering to enter the study. They may not be aware that after the screening process they may not be enrolled into the study, but they may have the opportunity to undergo another already approved treatment. The exclusion from being a trial participant does mean that they cannot be treated after all. Still, it is unclear how many patients in basket and umbrella trials do not enter the study because there are better treatment options for them and how many of them remain untreated due to the lack of any existing therapy.

## Conclusions

Basket and umbrella trials in oncology offer a new trial design aiming to test different types of treatments in an innovative and effective way. A new, flexible design, targeting single molecular alteration supposed to solve problems with recruitment and enable participation of patients with rare cancers turns out to be ethically challenged. An unusual flexibility and major differences in published results compared with the trials’ protocols, use of misleading terminology (e.g. “personalized medicine”), and complexity of malignancies are the examples of major concerns referring to ethically sensitive aspects: scientific validity, risk-benefit ratio and informed consent.

## Data Availability

The data used in this article are from publications available in the public domain.

## Abbreviations

AEs: Adverse events
FDA: Food and Drug Administration
Lung-MAP: Lung Cancer Master Protocol
N/R: Not reported
NCI-MATCH: National Cancer Institute - Molecular Analysis for Therapy Choice
OR: Objective response
OS: Overall survival
PD: Progressive disease
PFS: Progression-free survival
PFS6: Progression-free survival at 6 months of treatment
PR: Partial response
Pts: Patients
QoL: Quality of life
SCC: Squamous cell carcinoma
SCCA: Advanced squamous cell cancer
SD: Stable disease
SWOG: Southwest Oncology Group
TTP: Time to Progression
